# Flexible Sol-Gel—Processed Y_2_O_3_ RRAM Devices Obtained via UV/Ozone-Assisted Photochemical Annealing Process

**DOI:** 10.3390/ma15051899

**Published:** 2022-03-03

**Authors:** Hyeon-Joong Kim, Do-Won Kim, Won-Yong Lee, Kyoungdu Kim, Sin-Hyung Lee, Jin-Hyuk Bae, In-Man Kang, Kwangeun Kim, Jaewon Jang

**Affiliations:** 1 School of Electronic and Electrical Engineering, Kyungpook National University, Daegu 41566, Korea; dan7620@knu.ac.kr (H.-J.K.); ehdnjs5169@knu.ac.kr (D.-W.K.); yongsz@knu.ac.kr (W.-Y.L.); kkd9506@knu.ac.kr (K.K.); sinhlee@knu.ac.kr (S.-H.L.); jhbae@ee.knu.ac.kr (J.-H.B.); imkang@ee.knu.ac.kr (I.-M.K.); 2 School of Electronics Engineering, Kyungpook National University, Daegu 41566, Korea; 3 School of Electronics and Information Engineering, Korea Aerospace University, Goyang 10540, Korea; kke@kau.ac.kr

**Keywords:** sol-gel, Y_2_O_3_, RRAM, UV/ozone, photochemical, flexible

## Abstract

Flexible indium tin oxide (ITO)/Y_2_O_3_/Ag resistive random access memory (RRAM) devices were successfully fabricated using a thermal-energy-free ultraviolet (UV)/ozone-assisted photochemical annealing process. Using the UV/ozone-assisted photochemical process, the organic residue can be eliminated, and thinner and smother Y_2_O_3_ films than those formed using other methods can be fabricated. The flexible UV/ozone-assisted photochemical annealing process-based ITO/Y_2_O_3_/Ag RRAM devices exhibited the properties of conventional bipolar RRAM without any forming process. Furthermore, the pure and amorphous-phase Y_2_O_3_ films formed via this process showed a decreased leakage current and an increased high-resistance status (HRS) compared with the films formed using other methods. Therefore, RRAM devices can be realized on plastic substrates using a thermal-energy-free UV/ozone-assisted photochemical annealing process. The fabricated devices exhibited a resistive window (ratio of HRS/low-resistance status (LRS)) of >10^4^, with the HRS and LRS values remaining almost the same (i.e., limited deterioration occurred) for 10^4^ s and up to 10^2^ programming/erasing operation cycles.

## 1. Introduction

Owing to its extreme scalability, efficient power consumption, simple metal-active material–metal structure, and fast writing speed, resistive random access memory (RRAM) is considered a promising candidate for next-generation nonvolatile memory. The aforementioned attributes render RRAMs a solution to overcome the von Neumann bottleneck, allowing for the realization of a neuromorphic computing system that imitates the human brain [1,2,3]. For this imitation, memory devices with high density, high performance, and low-power operation are required. Several metal–oxide active channel materials, such as SiO_x_, ZrO_2_, TiO_x_, Hf_x_O, and Y_2_O_3_, have been considered for RRAM device fabrication [3,4,5,6,7,8,9,10,11,12]. Y_2_O_3_ is characterized by a high dielectric constant, large optical bandgap, and fast internal ion transport and is therefore considered quite attractive for use in such devices. Accordingly, it has also been considered as a high-k insulator to replace SiO_2_ in complementary metal–oxide-semiconductor processes [13,14]. Unfortunately, to realize RRAM arrays with single RRAM devices, the sneak path issue should be addressed. One solution involves the development of a one-transistor–one-RRAM unit structure [15,16]. Recently, Y_2_O_3_ has been used in thin-film metal–oxide-semiconductor transistors as a passivation layer, achieving the extreme external bias stability of the resulting transistors [17]. Therefore, the one-transistor–one-RRAM unit structure can be fabricated easily by simultaneously employing a Y_2_O_3_ layer as the passivation and active channel layers, yielding reduced processing times and costs. Conventional vacuum-based deposition methods have been used for depositing metal oxides, which is expensive and time-consuming [18,19,20,21]. These drawbacks have boosted the development of alternative deposition techniques, such as spin coating, sol-gel, and printing techniques, using solution-phase precursors [17,22,23,24]. The sol-gel technique is a simple approach for depositing high-quality, pure metal–oxide layers. By introducing two or three precursors, the structural, chemical, and electrical characteristics of the final metal–oxide products can be easily tuned. Furthermore, a liquid-phase precursor solution enables large-area applications, such as in inks for spin coating, dip coating, and printing techniques. These processes do not require expensive conventional vacuum-based deposition tools, thereby facilitating the low-cost fabrication of pure metal–oxide layers. However, to prepare high-quality, pure metal–oxide layers, a high-temperature annealing process (~500 °C) is required. Ultraviolet (UV)/ozone treatment is a promising alternative to the high-temperature annealing process. Generated oxygen radicals show high diffusive and reactive properties at low temperatures, promoting metal–oxide bonding during low-temperature annealing processes. The resulting flexible substrates can be employed in large-area flexible applications [25,26,27].

In this study, we investigated the influence of the UV/ozone-assisted photochemical annealing process on the structural, chemical, optical, and electrical properties of Y_2_O_3_. Thereafter, amorphous-phase Y_2_O_3_ films were prepared and deposited on substrates, which were thinner and exhibited lower surface roughness than the films fabricated using other methods. The decomposition of C-related groups afforded pure Y_2_O_3_ films without any thermal energy. For RRAM devices, sol-gel-processed Y_2_O_3_ active channel layers were successfully synthesized on plastic substrates without a thermal annealing process. The UV/ozone-assisted photochemical annealing process was used as an alternative to the conventional thermal annealing process. Ag/Y_2_O_3_/ITO flexible RRAM devices were successfully prepared on polyethylene terephthalate (PET) substrates using Ag and indium tin oxide (ITO) as the top and bottom electrodes, respectively, without any substrate deformation. The fabricated devices had conventional bipolar properties without any initial forming process. Therefore, the proposed fabrication process of amorphous-phase Y_2_O_3_-based RRAM devices provides a reference for the future application of flexible RRAM–based devices, such as wearable computers and soft robotics [28].

## 2. Materials and Methods

The sol-gel technique was used to form an ITO/Y_2_O_3_/Ag sandwich structure. Yttrium (III) nitrate tetrahydrate (99.99% trace metal basis; Sigma Aldrich) was used to synthesize the Y_2_O_3_ active channel layer. To prepare a solution precursor, 0.3 mol yttrium (III) nitrate tetrahydrate was dissolved in a 5 mL 2-methoxyethanol solution (anhydrous, 99.8; Sigma Aldrich). This mixture was ultrasonicated for 10 min, affording a clean and transparent solution precursor. A glass with an ITO deposited thereon was used as the substrate (surface resistivity: 70–100 Ω/sq, slide; Sigma Aldrich). Three such substrates were cleaned with acetone, isopropyl alcohol, and deionized water for 10 min each to remove any unwanted organic matter, dust, and other contaminants from the substrate. A nitrogen (N_2_) blow gun was used to remove excess liquid and dust from the cleaned substrates. The glass/ITO substrates were exposed to UV/ozone light (UV lamp wavelength: 254 nm; photon flux density: 16 mW/cm^2^; SEN Lights SSP16-110, Osaka, Japan) to eliminate any remaining organic contaminant and increase the surface energy. The distance between the sample and lamp was set to 3 cm. A small part of ITO was covered with Kapton tape to set a region for the electrical contact. The prepared substrates were coated with a thin layer of the precursors using the spin-coating technique (50 s at 3000 rpm). Three active channel layers (Films A, B, and C) were prepared to determine the effect of UV irradiation on each film. After the spin-coating process, Films A and B were baked on a hot plate (CORNING PC-420D, New York, NY, USA) for 10 min at 180 °C. Then, Film A was annealed in air for 2 h at 200 °C in a furnace (U1 Tech PTF-1203, Suwon, Korea). Film B was annealed in air for 2 h at 200 °C after being subjected to 2 h of UV irradiation. Film C was directly subjected to 8 h of UV exposure without baking or annealing after the spin-coating process (i.e., (UV)/ozone-assisted photochemical annealing process). Subsequently, 150 nm thick Ag top electrodes were formed on the Y_2_O_3_ films via high-vacuum thermal deposition at 5 × 10^−6^ torr. The deposition rate was 1.0 Å/s, and the top electrodes were adjusted to dimensions of 30 μm × 30 μm using a metal shadow mask. Flexible RRAMs were fabricated using the thermal-energy-free UV/ozone-assisted photochemical annealing process-based Y_2_O_3_ films on the PET substrates. Before deposition of the films, the surface was cleaned using the UV/ozone process. The Y_2_O_3_ films were deposited on ITO/PET substrates with dimensions of 2.5 cm × 2.5 cm (Sigma Aldrich). The thermal-energy-free process was used to deposit the Y_2_O_3_ films to minimize the deformation of the ITO/PET substrate.

The characteristics of the RRAM devices were evaluated using multiple measurement methods. The phase and structural characteristics of the Y_2_O_3_ active channel layer were evaluated using grazing incidence X-ray diffraction (GIXRD; Philips X’pert Pro, Philips, Amsterdam, The Netherlands) using CuKα radiation at a wavelength of 1.54 Å and a small incident angle of 0.3°. X-ray photoelectron spectroscopy (XPS; ULVAC-PHI, Chigasaki, Japan) was used for the chemical analysis of the Y_2_O_3_ oxide layer. Moreover, the surface roughness of the Y_2_O_3_ layer was assessed, and the film thickness was analyzed using scanning probe microscopy (SPM; Park NX20, tapping mode, Suwon, Korea) and field emission scanning electron microscopy (Hitachi 8230, Tokyo, Japan). UV–visible (UV–vis; LAMBDA 265, MA, USA was used for the optical analysis of the coated precursor and annealed oxide film. Furthermore, a semiconductor parameter analyzer (Keithley 2636B, Keithley Instruments, Cleveland, OH, USA) and a probe station (MST T-4000A, Hwaseong, Korea) were used to measure the electrical performances of the Ag/Y_2_O_3_/ITO-structured RRAM devices. To analyze the endurance characteristics of the Ag/Y_2_O_3_/ITO-structured RRAM devices, the current visible pulse voltage stress test was employed instead of the current–voltage (I–V) sweep method [24].

## 3. Results and Discussion

**Figure 1:**
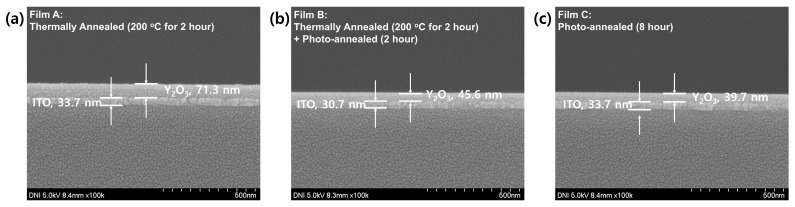
Cross-sectional scanning electron microscope images of Y_2_O_3_ films obtained under different annealing treatments: (**a**) thermally annealed films, (**b**) thermally and photochemical annealed films, and (**c**) photochemical annealed films, respectively.

Figure 1 shows the cross-sectional scanning electron microscopic (SEM) images of the fabricated devices obtained under different annealing conditions. The thickness of the Y_2_O_3_ film annealed at 200 °C was ~70 nm, almost twice that of the films obtained via the UV/ozone-assisted photochemical annealing process (~40 nm). This suggests that the UV/ozone-assisted photochemical annealing process can aid in the organic volatilization and decomposition of the films. Furthermore, the surface morphology of the films obtained via this process was more uniform and smoother than that of the films obtained via other methods.

**Figure 2:**
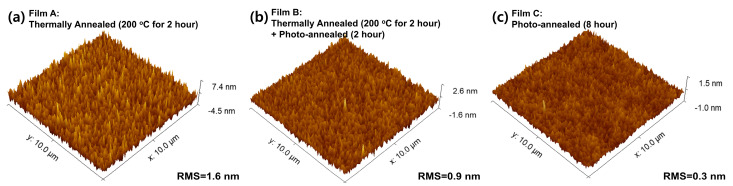
Scanning probe microscopy images of the Y_2_O_3_ films with different annealing treatments: (**a**) thermally annealed films, (**b**) thermally and photochemical annealed films, and (**c**) photochemical annealed films, respectively.

Figure 2 shows the three-dimensional SPM images of the Y_2_O_3_ films obtained under different annealing treatments. The root-mean-square (RMS) roughness values of films A, B, and C were 1.6, 0.9, and 0.3 nm, respectively. This indicates that the UV/ozone-annealed Y_2_O_3_ thin films were smoother than the other films, as confirmed by the reduced surface roughness. Using the UV/ozone-assisted photochemical annealing process, the organic residue can be removed based on the formation of oxygen radicals during the process, affording very smooth Y_2_O_3_ films with low RMS roughness values.

**Figure 3:**
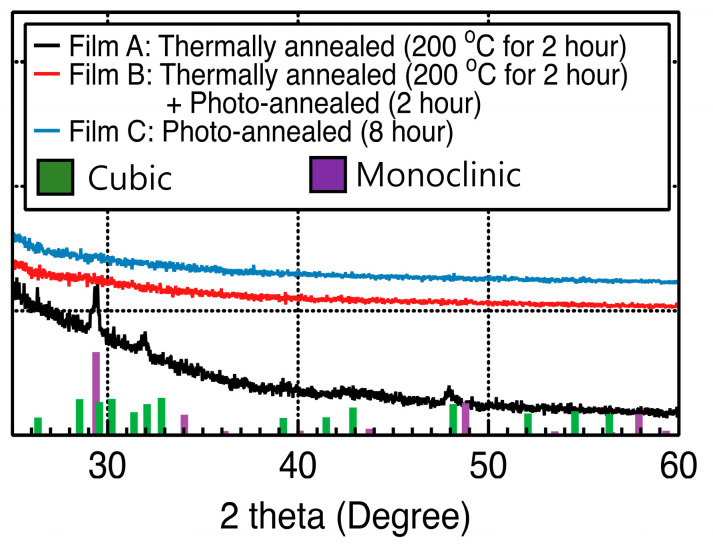
Grazing incidence X-ray diffraction spectra of Y_2_O_3_ films obtained under different annealing treatments.

Figure 3 shows the GIXRD spectra of the Y_2_O_3_ films obtained under different annealing treatments. The films annealed at 200 °C showed peaks at 29.4°, 32.4°, and 48.2°, indicating the polycrystallinity of these films, which did not undergo the UV/ozone-assisted photochemical annealing process. The films consequently showed a mixture of cubic (JCPDS 41-1105) and monoclinic (JCPDS 44-0399) Y_2_O_3_. At <400 °C, cubic Y_2_O_3_ was stable; however, metastable monoclinic Y_2_O_3_ was also detected at these temperatures. The crystallite size of the Y_2_O_3_ films was determined using the Scherrer equation with the full width at the half maximum of the main diffraction peak at 32.4° for cubic Y_2_O_3_ and at 29.4° for monoclinic Y_2_O_3_.

D = (0.9 λ)/(β cos θ)(1)

where D, λ, β, and θ indicate the crystalline size, X-ray wavelength (1.54 Å), line broadening at the half maximum of each diffraction peak of the pattern, and peak position (Bragg angle), respectively.

The crystalline sizes of the cubic and monoclinic Y_2_O_3_ structures were calculated to be 20 and 25 nm, respectively. However, no sharp diffraction peaks were detected in the case of Y_2_O_3_ films subjected to the UV/ozone-assisted process, indicating that all formed films comprised amorphous-phase Y_2_O_3_. Based on the SEM images, the Y_2_O_3_ film formed using the UV/ozone-assisted photochemical annealing process was thinner than that obtained via the single annealing process at 200 °C. The former films were denser and therefore thinner than the latter films. The crystalline size of a film is typically inversely proportional to its thickness [29,30].

**Figure 4:**
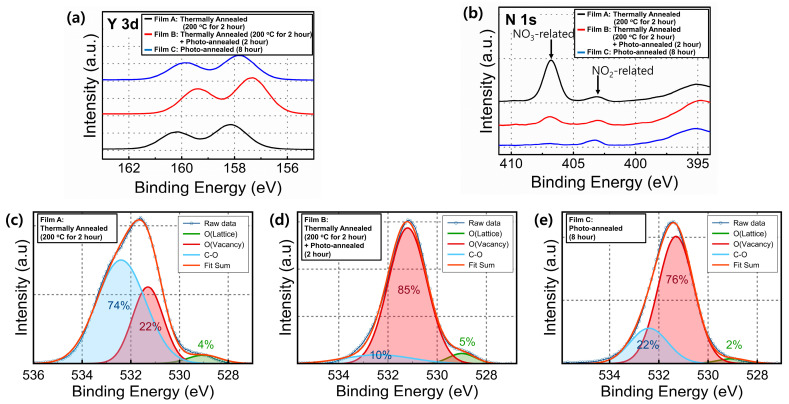
(**a**) Y 3d, (**b**) N 1s, and (**c**–**e**) O 1s XPS spectra of the sol-gel-processed Y_2_O_3_ films obtained under different annealing treatments.

The chemical composition of the Y_2_O_3_ films obtained under different annealing treatments was investigated using XPS. The obtained data were calibrated based on the peak position of C 1s (284.5 eV). Figure 4a shows the Y 3d XPS spectra of the films. The spectra showed two peaks that corresponded to two splitting orbitals (Y 3d_5/2_ and Y 3d_3/2_ at 156.0 and 158.7 eV, respectively). Irrespective of the annealing condition, the Y 3d_5/2_ and Y 3d_3/2_ peaks shifted toward higher binding energy compared with the stoichiometric Y_2_O_3_ oxide [31,32]. As shown in the N 1s XPS scan (Figure 4b), the intensity of the NO_3_^−^-related signal decreased after the UV/ozone-assisted photochemical annealing process. However, the intensity of the NO_2_^−^-related signal remained almost unchanged with/without this process. Figure 4c–e show the XPS spectra for the O 1s core level of the Y_2_O_3_ films. The obtained data were deconvoluted into three peaks at 529, 531.4, and 532.1 eV, corresponding to oxygen ions combined with metal cations (O_L_), surface physisorbed oxygen, and C–O binding, respectively [33]. The amount of physisorbed oxygen was higher for the films annealed using the UV/ozone-assisted photochemical annealing process than for those not subjected to annealing. Moreover, the decreased C–O binding proportion indicated that the process can aid in the organic volatilization and decomposition of the films. This observation was also consistent with the results of the cross-sectional SEM and SPM data obtained for the Y_2_O_3_ films subjected to the UV/ozone-assisted photochemical annealing process.

**Figure 5:**
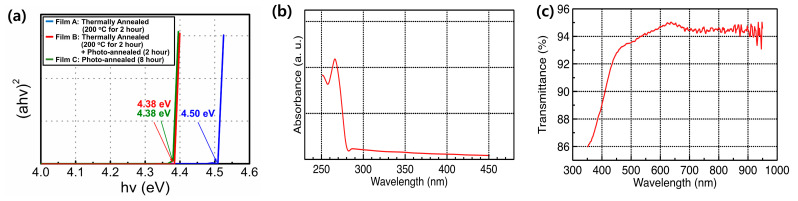
(**a**) Optical bandgap of Y_2_O_3_ films obtained under different annealing treatments; (**b**) light-absorption characteristics of Y(NO_3_)_3_·4H_2_O on a glass substrate; (**c**) transmittance spectra of ITO/glass substrate.

Figure 5 shows the optical transmittance and optical bandgap of the Y_2_O_3_ films obtained under different annealing treatments. The extracted optical bandgap was estimated by extrapolating the line segment in Figure 5a. The following equation was used.

(2) $${\left(\alpha h\upsilon \right)}^{1/n}=A\left(h\upsilon -E_{g}\right),$$

where *α* denotes the absorption coefficient, *n* is 0.5 for direct transition, *A* is a constant, and *E_g_* is the optical bandgap.

Bandgap values of 4.50, 4.38, and 4.38 eV were obtained for films A, B, and C, respectively. After the solvent-drying process at 150 °C, the absorption spectra of Y(NO_3_)_3_·4H_2_O on the glass substrate revealed that strong light absorption occurred below 280 nm (Figure 5b). A UV lamp with a wavelength of 254 nm facilitated the photochemical activation of Y(NO_3_)_3_·4H_2_O via the UV/ozone process [25]. Figure 5c shows the transmittance spectra of ITO/glass substrate.

**Figure 6:**
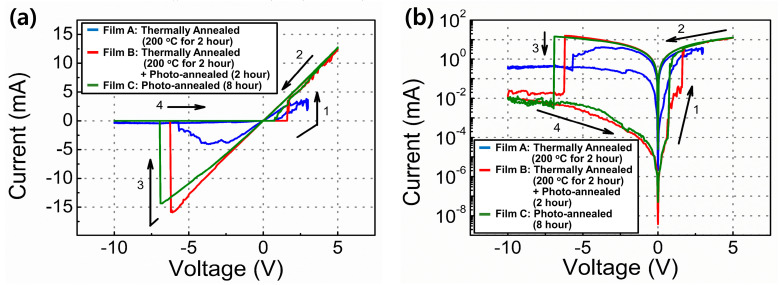
Representative I–V curves of ITO/Y_2_O_3_/Ag RRAM devices. The arrows and numbers indicate the voltage bias directions. (**a**) Linear scale and (**b**) log scale.

Figure 6 shows the I–V curves measured for the RRAM devices fabricated under different annealing treatments. Each device exhibited the properties of conventional bipolar RRAM. Furthermore, for all the devices, no process was required for the formation of the initial conductive path. RRAM devices comprising oxygen-vacancy-defect-rich materials or devices based on the electrochemical metallization (ECM) mechanism with chemically active top electrodes (such as Ag and Cu) exhibited a forming-process-free operation. In this system, the deposited top Ag electrode served as the main source for the formation of a conductive path between the top and bottom ITO electrodes. Our previous study demonstrated that using inert top Au electrodes, the fabricated device showed no resistive switching memory properties, indicating that the fabricated RRAM device was a type of ECM RRAM [11]. The initial resistance status was a high-resistance status (HRS). When the positive voltage was biased, the resistance decreased abruptly at ~+2.0 V, entering the low-resistance-status (LRS) regime. The voltage corresponding to changes in the resistance status from HRS to LRS is referred to as the SET voltage. In contrast, when the negative voltage was biased, the resistance increased abruptly at ~−6.0 V, entering the HRS regime. The voltage corresponding to changes in the resistance status from LRS to HRS is referred to as the RESET voltage. When positive voltage is biased at the top Ag electrodes, Ag ions (Ag^+^) can be formed at the interface between Y_2_O_3_ and these electrodes via an oxidation process (Ag → Ag^+^ + e^−^). The Ag^+^ can move to the bottom electrodes via Y_2_O_3_ films. The Ag^+^ was then reduced to Ag atoms (Ag^+^ + e^−^ → Ag) again. When the accumulated Ag atoms formed a conductive path between the bottom and top electrodes, the current increased abruptly at the SET voltage. When the negative voltage was biased at the top Ag electrodes, the previously formed Ag conductive paths were ruptured by the reduction process at the interface between Y_2_O_3_ and the electrodes. The current decreased abruptly again at a RESET voltage. The resistance status of the UV/ozone-assisted photochemical process-based Y_2_O_3_ RRAM devices changed abruptly. However, the resistance states of the RRAM devices consisting of the Y_2_O_3_ films without the UV/ozone process changed gradually.

**Figure 7:**
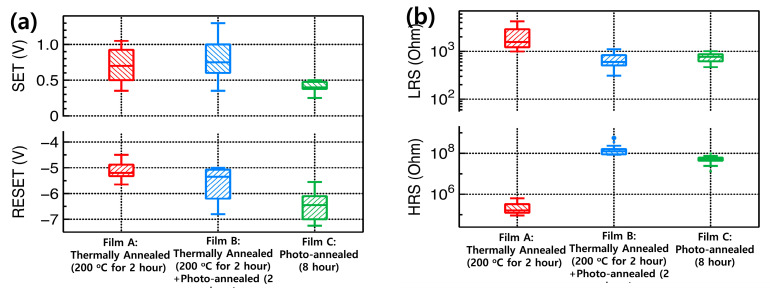
Extracted performance parameters of the ITO/Y_2_O_3_/Ag RRAM devices: (**a**) SET and RESET voltages; (**b**) LRS and HRS values.

The extracted SET voltage, RESET voltage, LRS, and HRS (from 10 devices) are plotted in Figure 7 to determine the statistical distribution. In all cases, the RESET voltage was larger than the SET voltages. The RESET voltages changed significantly, owing to the voltage ramp rate (V/s) [34,35,36]. Here, when the ramp rate of the RESET was fast, the RESET voltage was high. The HRS/LRS ratio of the UV/ozone-assisted Y_2_O_3_-based RRAM devices (over 10^4^) was larger than that of the other devices because the current decreased significantly under HRS. Based on the GIXRD and XPS data, the UV/ozone-assisted Y_2_O_3_ films consisted of an amorphous phase and were successfully converted into pure Y_2_O_3_ films. The disordered structure of this phase can suppress the carrier mobility. However, for crystallized films, the increased grain-boundary size can enhance the carrier mobility inside the films, leading to leakage current associated with the HRS. This resulted in a decreased leakage current between the two electrodes and higher HRS values in the case of the UV/ozone photochemical annealing process-assisted Y_2_O_3_-based RRAM devices compared with those of other devices [37,38,39].

**Figure 8:**
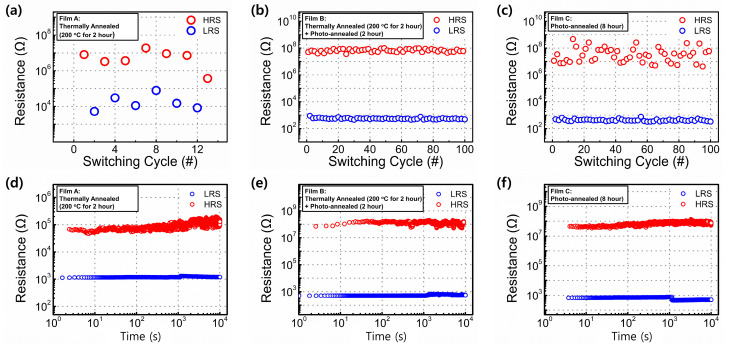
(**a**–**c**) Representative endurance properties; (**d**–**f**) retention properties of the ITO/Y_2_O_3_/Ag RRAM devices obtained under different annealing treatments.

The nonvolatile memory characteristics of the fabricated ITO/Y_2_O_3_/Ag RRAM devices were evaluated based on the endurance and retention data (Figure 8) of each device. The HRS and LRS values were measured at +0.1 V after programing (+5.0 V for 50 ms) and erasing (−10.0 V for 50 ms) operations. The HRS and LRS values remained approximately the same (i.e., limited deterioration was observed) regardless of the annealing treatment. However, thermally annealed Y_2_O_3_-based RRAM devices exhibited poor endurance properties (significant deterioration occurred after only 10 cycles). Table 1 shows a comparison of the resistive switching characteristics of low-temperature RRAM devices.

**Figure 9:**
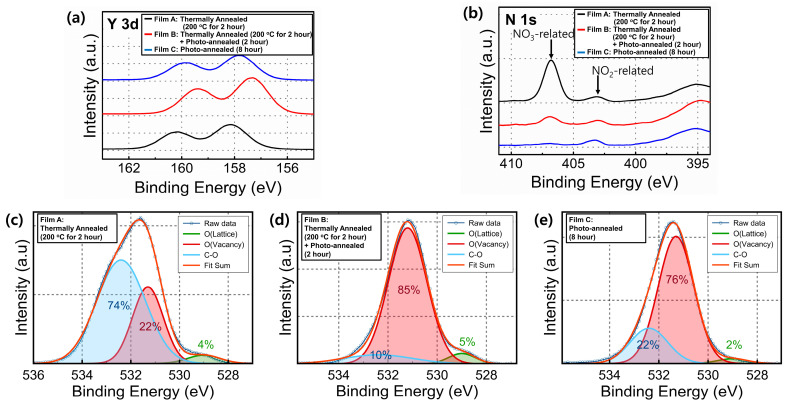
(**a**) Optical image of a fabricated flexible ITO/Y_2_O_3_/Ag RRAM device. Inset shows a schematic of the fabricated devices. (**b**) Representative I–V curve in the log scale. Inset shows an I–V curve in the linear scale. Arrows and numbers indicate the voltage bias directions, (**c**) endurance, and (**d**) retention properties of the devices.

Using the thermal-energy-free UV/ozone-assisted photochemical annealing process, flexible RRAM devices can be prepared on plastic substrates. Figure 9a,b show an optical image and the representative I–V curves of the fabricated flexible RRAM devices. The ITO/Y_2_O_3_/Ag devices fabricated on PET exhibited forming-process-free and conventional bipolar RRAM properties. The HRS/LRS ratio was ~10^4^. Furthermore, the HRS and LRS values remained almost the same (i.e., limited deterioration was observed) for 10^4^ s and for up to 10^2^ programming/erasing operation cycles with minimal degradation (Figure 9c,d).

## 4. Conclusions

ITO/Y_2_O_3_/Ag RRAM devices were successfully fabricated using a thermal-energy-free UV/ozone-assisted photochemical annealing process. This process can eliminate the organic residue and form thinner and smother Y_2_O_3_ films compared with those obtained using other methods. Further, the pure and amorphous-phase Y_2_O_3_ films formed using the aforementioned process achieved a decreased leakage current. Using the suggested thermal-energy-free UV/ozone-assisted photochemical annealing process, RRAM devices can be fabricated on plastic substrates. The flexible ITO/Y_2_O_3_/Ag RRAM devices prepared on PET substrates exhibited conventional bipolar properties and required no forming process. Moreover, almost the same HRS and LRS values were retained (i.e., minimal deterioration was observed) for 10^4^ s and up to 10^2^ programming/erasing operation cycles.

## Figures and Tables

**Table 1:** Comparison of the resistive switching characteristics of the low-temperature-processed RRAM devices.

Reference	Material System	Process Temperature (°C)	V_SET_/V_RESET_	HRS/LRS	Forming	Endurance (Cycle)/Retention (s)	Conductive Filament Type
[40]	Au/Graphene/SnO_2_	80	+2.5 V/+1.0 V	~10	No	~1 × 10^2^/1.8 × 10^3^	Oxygen vacancy
[41]	ITO/ZrO_2_/W	45	+1.0 V/−1.5 V	~10	No	~2 × 10^2^/10^4^	Oxygen vacancy
[42]	Al/Pt/AlO_x_/Ni	300	+2.5 V/−0.75 V	~10	No	~1 × 10^2^/~10^4^	Oxygen vacancy
[43]	P^+^/SiN_x_/N^+^–Si	300	+3.0 V/−1.5 V	~10^2^	Yes	~10^5^/~10^4^	Nitride vacancy/ Si dangling bonds
This work	Ag/Y_2_O_3_/ITO	RT	+0.5 V/−7.0 V	~10^4^	No	10^2^/10^4^	ECM

## Data Availability

Data available in a publicly accessible repository.
